# Probing Cellular Dynamics with a Chemical Signal Generator

**DOI:** 10.1371/journal.pone.0004847

**Published:** 2009-03-16

**Authors:** Brandon Kuczenski, Warren C. Ruder, William C. Messner, Philip R. LeDuc

**Affiliations:** 1 Department of Mechanical Engineering, Carnegie Mellon University, Pittsburgh, Pennsylvania, United States of America; 2 Department of Biomedical Engineering, Carnegie Mellon University, Pittsburgh, Pennsylvania, United States of America; Emory University, United States of America

## Abstract

Observations of material and cellular systems in response to time-varying chemical stimuli can aid the analysis of dynamic processes. We describe a microfluidic “chemical signal generator,” a technique to apply continuously varying chemical concentration waveforms to arbitrary locations in a microfluidic channel through feedback control of the interface between parallel laminar (co-flowing) streams. As the flow rates of the streams are adjusted, the channel walls are exposed to a chemical environment that shifts between the individual streams. This approach can be used to probe the dynamic behavior of objects or substances adherent to the interior of the channel. To demonstrate the technique, we exposed live fibroblast cells to ionomycin, a membrane-permeable calcium ionophore, while assaying cytosolic calcium concentration. Through the manipulation of the laminar flow interface, we exposed the cells' endogenous calcium handling machinery to spatially-contained discrete and oscillatory intracellular disturbances, which were observed to elicit a regulatory response. The spatiotemporal precision of the generated signals opens avenues to previously unapproachable areas for potential investigation of cell signaling and material behavior.

## Introduction

Many aspects of cell function can be described as generalized feedback systems which monitor conditions in and around the cell and respond by modulating biochemical pathways. The workings of these mechanisms can be exposed by deliberately introducing a precise change to the cell's environment and observing the response of the cellular subsystem. Recent investigations have used varying chemical environments to probe the dynamic characteristics of signaling networks [1], [2] and gene regulation [3]. Interpretation of the responses in the context of signal-processing and feedback can generate insight into the regulatory behavior of cells [1].

The regulation of cytosolic calcium (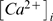) is one research area in which observation of cellular response to dynamic signals has proven fruitful. Eukaryotic cells maintain a cytosolic calcium concentration many orders of magnitude lower than the surrounding environment by constantly pumping calcium into a high-concentration store in the sarco-endoplasmic reticulum, and out of the cell across the plasma membrane. Messenger molecules can trigger the release of calcium from the store, a phenomenon which is implicated in a wide array of cellular processes [4]–[6]. The cell's mechanisms for releasing and re-sequestering calcium in the store respond to feedback from a variety of sources, including calcium concentration itself on either side of both membranes [7], [8]. Because calcium signaling events occur across a wide spatiotemporal range and in the context of a complex feedback network, analysis of calcium regulation demands fine control of biological conditions and measurement of cellular responses.

Microfluidic techniques offer improved control of cellular environments and have seen a broad and increasing role in cell biology research in recent years. Cells can be cultured in microfluidic devices, permitting long-term monitoring in a carefully controlled environment [3], [9], [10]. Techniques have been demonstrated that expose cells to microfluidic “switching flows” which permit bulk changes in cellular surroundings at a faster rate than traditional perfusion [11], [12]. The small dimensions of microfluidic channels lead to a condition of laminar flow which can be exploited to situate two dissimilar fluid streams parallel to one another in a channel [13]–[15]. Such co-flowing streams have been used to concurrently treat distinct regions of cells, clusters of cells or embryos with multiple fluid environments that differ in temperature or chemical makeup [16]–[18]. Hydrodynamic focusing, where one fluid stream is tightly constrained between two others, has also been used to deliver solutes selectively to subpopulations of cells [19]. Photolysis can be used to release caged chemicals into cells' interior or exterior environments with high spatial specificity [20]. Flow photolysis combines photolysis with microperfusion and has the capacity to expose cells to minute chemical perturbations [21]. However, these techniques offer limited ability to vary the extracellular environment continuously in time. Photolysis also requires the manufacture and use of caged molecules and elaborate optics.

We present a technique that affords continuous variation of the extracellular environment through the measurement and feedback control of pressure in the fluid pumping mechanism. This refinement allows inlet pressures to be adjusted accurately and continuously, and can be used to control the lateral position of the interfacial plane between co-flowing streams [22]. Measurement of inlet pressures provides a mechanism to estimate the position of the interfacial plane with high spatiotemporal precision. Below, we demonstrate that this estimate accurately reflects the fluid conditions inside the channel, and that by adjusting the position of the interface, time-varying chemical signals can be generated and applied to living cells, and their responses observed.

We demonstrate our technique in observation of NIH 3T3 fibroblasts exposed to ionomycin, a calcium ionophore, in a time-varying fashion. Ionophores are molecules which permit ion traversal of lipid membranes which are ordinarily ion-impermeable [23]. Calcium ionophores can enter the cell through the plasma membrane to effect a rapid depletion of cells' internal calcium stores, and have long been used to probe the dynamics of calcium regulation in many different cell lines [24]–[27]. However, the response of non-excitable cells to brief (1–3 s) ionophore exposure has not been documented. We exposed intact fibroblasts to precisely time-varying concentrations of ionomycin, a calcium ionophore, while monitoring the concentration of 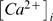 using Fluo-4 AM, a membrane-permeable fluorescent calcium indicator. The cellular responses to discrete and oscillatory exposure point to novel insights in the spatiotemporal dynamics of calcium signaling.

## Results

### Varying the Cellular Environment

**Figure 1:**
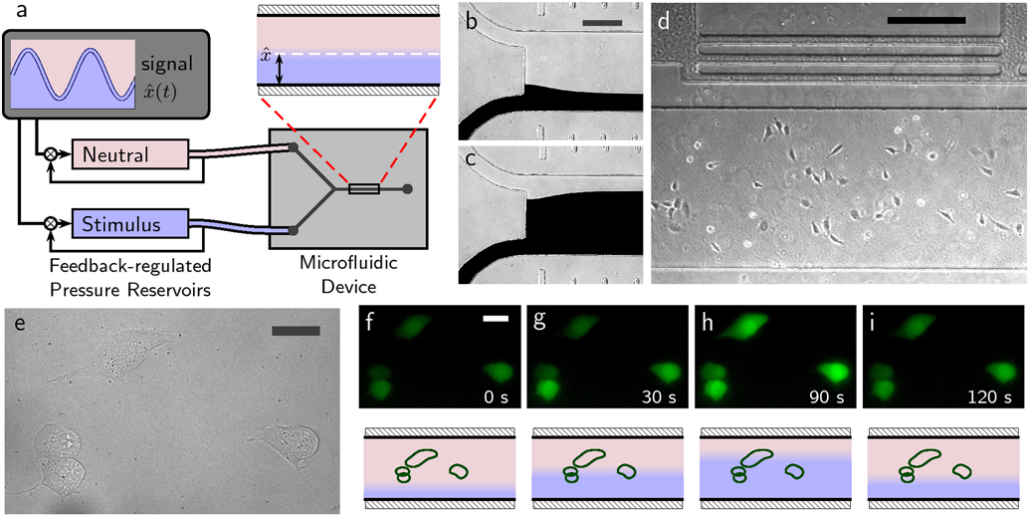
Generating chemical signals in a microfluidic device using pressure feedback. (a) A schematic of device operation. Pressure commands from a supervisory controller direct two independent pressure regulators. The regulators operate on two fluid reservoirs, one containing a neutral immersion fluid (Ringer's solution containing 1 µM Ca2+) and the other containing a stimulus stream (identical Ringer's solution plus 1 µM ionomycin), which feed the inlets to a microfluidic device. Changes in static pressure cause the interface in the outlet channel to shift. (b–c) Two different interface positions, visualized using water (top stream) co-flowing with watercolor ink (bottom stream). Scale bar is 250 µm. (d) Cells cultured in an outlet channel. The horizontal bars along the top of the image indicate the distance from the point of confluence (longitude). Scale bar is 250 µm. (e–i) Cells responding to a shifting interface. Scale bars are 25 µm. (e) DIC image of four cells. (f–i) Fluorescence images of the cells at t = 0, 30, 90, and 120 s, accompanied by cartoons indicating relative interface position.

Through the use of feedback-controlled laminar flow we were able to continuously vary the chemical environment surrounding target cells cultured in a microfluidic channel (Fig. 1). We used microfluidic devices with two inlet channels of equal fluid resistance which merged to form a single outlet channel. A laminar interface developed in the outlet channel as long as there was positive flow into both inlets, and the position of the interface depended on the relative flow rates into each inlet. Because the flow rate through a channel of fixed geometry depends only on the pressure drop across the channel, feedback control of inlet pressures permitted immediate control over interface position in the outlet channel. The sensitivity of the interface position to changes in pressure is determined by the ratio of the fluid resistance of the inlet and outlet channels, and is thus a property of the microfluidic device design.

Figure 1a summarizes the operation of the experimental apparatus. The static pressures of two fluid reservoirs were controlled by a pair of prototype feedback pressure regulators according to commands received from a supervisory controller [22]. By adjusting the pressures at the inlets the interface was made to shift laterally (Fig. 1b–c). During experiments, the fluid pressure in each reservoir was automatically measured at the time of each fluorescent image capture. Measurements of reservoir pressures were used to estimate the position of the interface in the channel. Although flow rates through each inlet varied with time, the total flow rate through the outlet channel was kept constant, maintaining a steady velocity profile in the outlet channel. Impingement of cells upon the fluid flow was judged not to induce mixing nor to have a significant effect on the velocity profile (see Supplementary Material S1).

Figure 1d shows a survey image that can be used to determine a target cell's location, measured as “latitude” and “longitude.” Latitude is dimensionless and represents location across the channel width. Longitude represents distance downstream of the point of confluence. Coded marks along the edges of the channel indicate the longitude in mm. The image shown in Fig. 1d is from a region 7–8 mm downstream from the confluence point.

Cells cultured in the microfluidic device were recruited as indicators of the chemical environment in the channel. When the environment surrounding the cells is absent of ionophore, the cells can be expected to maintain a steady state; under exposure to ionophore, the cells can be expected to signal this condition in a positively observable way, by an increase in fluorescent intensity due to heightened 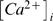. Fluorescent intensity of a target cell should thus be indicative of the location of the laminar interface relative to that of the cell.

To verify that interface position had an effect on cellular environment, we loaded cells cultured in a microfluidic device with a fluorescent calcium indicator. We prepared two fluid reservoirs, one with a neutral imaging fluid (Ringer's solution containing 1 µM Ca^2+^) and the other with a stimulus fluid (identical Ringer's solution plus 1 µM ionomycin). The fluid reservoirs were loaded into the pressure regulators and attached to the microfluidic device. The cells were monitored on a microscope during pressure regulator operation.

Modulation of fluid flows was observed to effect an immediate, reversible change in mean cell-wide fluorescent intensity. Through careful control of inlet pressure, cells could be selectively exposed to the ionophore depending on their lateral position in the channel (Fig. 1e–i and Supplementary Video S1).

### Cellular Responses to Stimulus

**Figure 2:**
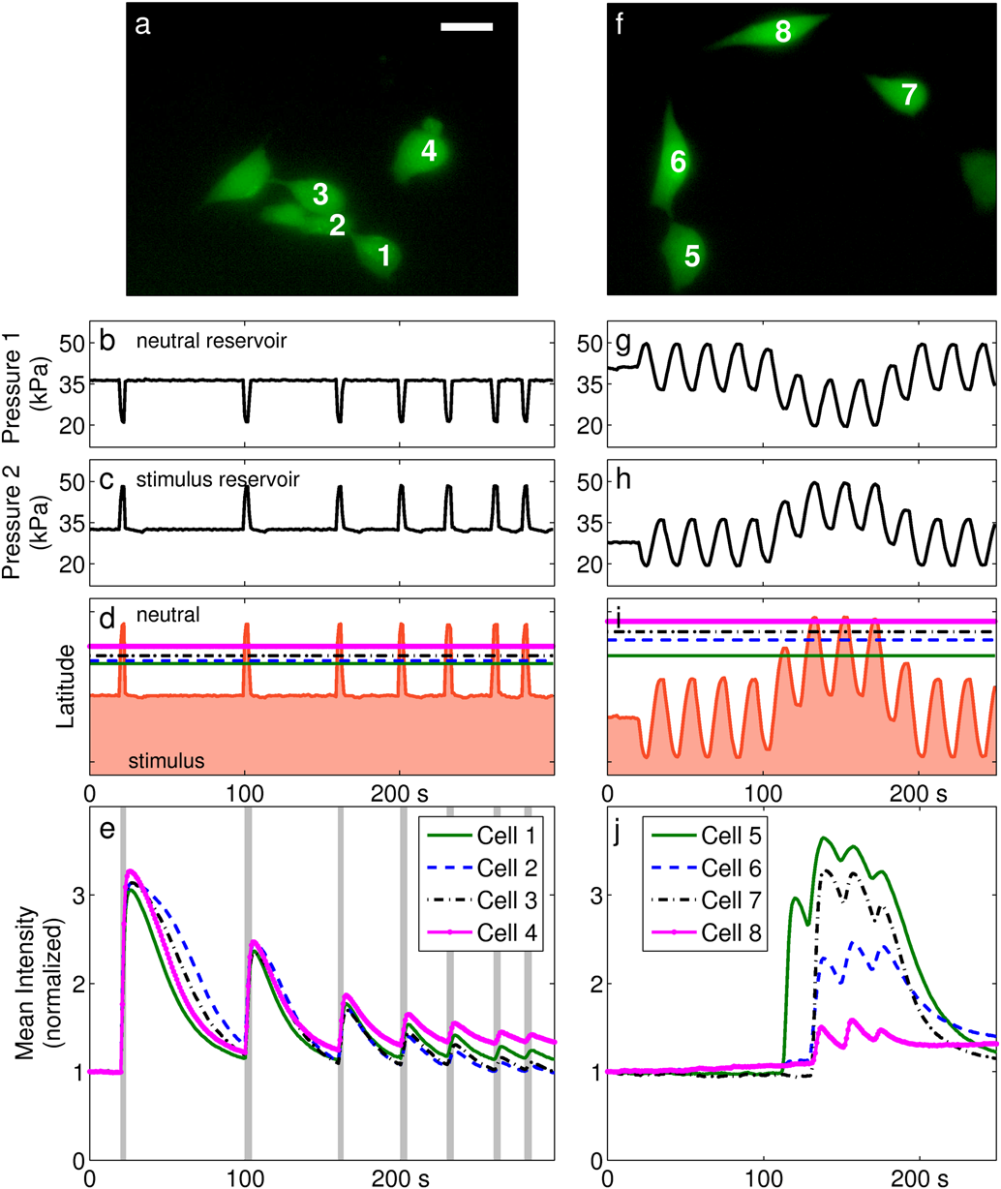
Cellular responses to stimulus. Two different clusters of cells were loaded with Fluo-4 AM and exposed to ionomycin through modulation of interface position. (a,f) Fluorescent micrographs showing the cells under observation. Scale bar is 25 µm. (b,g) Measurements of static pressure in the reservoir containing the neutral stream. (c,h) Pressure measurements from the reservoir containing the stimulus stream. (d,i) An estimate of interface position across the width of the channel as a function of time as determined from pressure measurements. Units are dimensionless latitude. The horizontal lines indicate the centroids of the regions of interest labeled in (a,f). (e,j) Mean fluorescent intensity over each region of interest vs. time, normalized to the initial value. Vertical shading in (e) indicates periods when cells were predicted to be under maximal exposure. Data from (f–j) are shown in Supplementary Video S2.

Figure 2 shows the results of two typical experiments in which clusters of cells were repeatedly exposed to the stimulus for short periods. In all experiments, the onset of stimulus could be predicted through comparison of cell position to estimated interface position. These results demonstrate that the model for interface position reflects the actual chemical conditions inside the channel, and that reservoir pressures can be used effectively as a proxy for direct measurement of the interface position if the geometry of the microfluidic device is known.

Cells were observed to respond rapidly to the presence of the ionophore, manifesting a multifold increase in mean fluorescent intensity within 1–3 seconds of the interface crossing the target cell's latitude. Under prolonged stimulus, fluorescent intensity reached a maximum after 20–50 s and then began a recovery towards baseline levels. If the stimulus was removed before this time, the recovery to baseline levels was more rapid.

Each cell's location in the channel was first determined from a low-magnification survey image (not shown; see Fig. 1d). The cells (Fig. 2a,f) were then monitored at 1 s intervals while the pressures in the reservoirs (Fig. 2b–c,g–h) were modulated. Inlet reservoir pressures were measured automatically at the moment of each fluorescent image capture. The position of the interface was estimated from pressure measurements and compared to estimates of the cells' latitudes (Fig. 2d,i). These interface position measurements could then be correlated to measurements of fluorescent intensity. The data shown in Fig. 2e,j are fluorescent intensity vs. time over the regions of interest labeled in Fig. 2a,f. Data from Fig. 2f–j are shown in Supplementary Video S2.

The two experiments shown in Figure 2 are representative of a total of 50 experiments in which a total of 166 cells were observed. Of these, 131 showed a distinguishable response to stimulus with ionomycin. From the data it is evident that cells experience a change in their surrounding environments when the laminar interface is made to cross their position. Small subpopulations of cells respond uniformly and synchronously to recurrent brief stimuli, and the fluorescent intensity appears to increase when the cells are under stimulus and decrease when the stimulus is discontinued. In addition, repeated or prolonged stimulus can be seen to exhaust the cell's capacity to produce a response. This observation is consistent with the hypothesis that extracellular ionomycin enters the cells and motivates transport of calcium out of internal stores [26], [28].

### Spatiotemporal Precision

**Figure 3:**
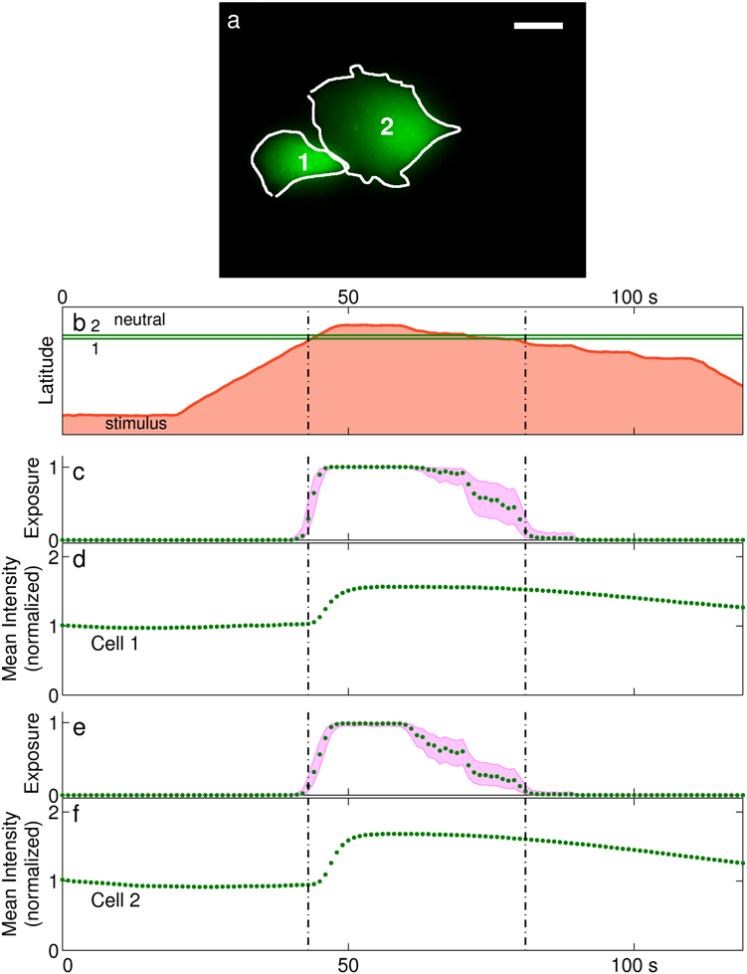
Assessment of spatiotemporal precision. (a) Two cells located close to the top of a channel. The regions of interest used to measure fluorescent intensity are marked. Scale bar is 25 µm. (b) Interface position vs. time, as determined from measurements of inlet pressure. The two horizontal lines mark the centroids of the regions of interest in panel a. Vertical lines delineate the period of exposure. (c–f) The estimated exposure of each cell to stimulus and its response, vs. time. (c,e) show estimated exposure to stimulus, normalized by the cell's area and the maximal concentration. Shaded areas represent standard error of the interface position estimate. (d,f) show mean fluorescent intensity over the cell's area, normalized to the initial value.

Feedback control of reservoir pressures enables accurate adjustment of the relative flow rates of the two streams, which determine the position of the interface at the point of inlet channel confluence. Comparison of the estimated interface position and the cell response can be used to evaluate device precision (Fig. 3). Because interface position is not measured directly, this evaluation cannot extract the exact time or positional resolution of the apparatus. However, if an increase in a cell's fluorescent intensity is taken to be a sign of exposure to ionophore, then the cell can act as an indicator of the actual position of the interface. If the interface is made to transit the cell quickly and completely, then the time delay between the predicted transit and the observed response provides an upper bound for the time precision of the apparatus. Similarly, by making the expected position of the interface transit the cell slowly, the spatial difference between actual and predicted interface position can be observed.

The time required for changes in reservoir pressure to manifest as increases in fluorescent intensity appears to be on the order of a single data point (1 s) or faster (Fig. 2a–e). The expected transport delay for reservoir pressure changes to impinge on cells as an interface shift depends on the cells' longitude versus the fluid velocity in the channel and was on the order of 50–250 ms in our study. The observed time precision is of the same order as this transport delay.

The precision of the interface position estimate depends on accurate predictions of the inlet flow rates, and thus on accurate measurements of the fluid resistance of the inlet and outlet channels. Because the actual interface position depends on the ratio of flow rates, the error in interface position is proportional to channel width. For the present apparatus, the cumulative standard error of measurement of interface position was found to be 1.8% of channel width, or about 9 microns for the devices used. Most of this error is attributable to defects in microfluidic device manufacture (see Supplementary Material S1).

Once a cell's location in the channel is known, its exposure to stimulus can be computed based on the interface position. The concentration of the stimulus in the outlet channel depends on its diffusion into the neutral stream, which causes the concentration gradient across the interface to broaden with increasing longitude. The concentration profile was estimated based on numerical and empirical models of diffusion between laminar streams (see [29], [30], Supplementary Material S1). A target cell's exposure to stimulus was determined by integrating the concentration profile over a region approximating the cell's area and dividing by the area. This resulted in a normalized estimate of exposure to stimulus as a function of interface position.

In the experiment shown in Fig. 3, the interface was made to sweep gradually across a pair of cells located close to the top of the channel (Fig. 3a–b). Each cell's exposure to stimulus was estimated based on its distance from the interface (Fig. 3c,e). The exposure estimates can be compared with observations of fluorescent intensity (Fig. 3d,f). The error in the exposure estimate can be visualized by offsetting the cell's latitude in either direction by the standard error in interface position when computing exposure (shaded areas on plots). Exposure can be made more precise in time by moving the interface at higher speed, as in the rising edge of the stimulus curve. More gradual motions and finer control of chemical exposure are subject to greater uncertainty, as in the falling edge of the stimulus.

### Chemical Signal Generation

**Figure 4:**
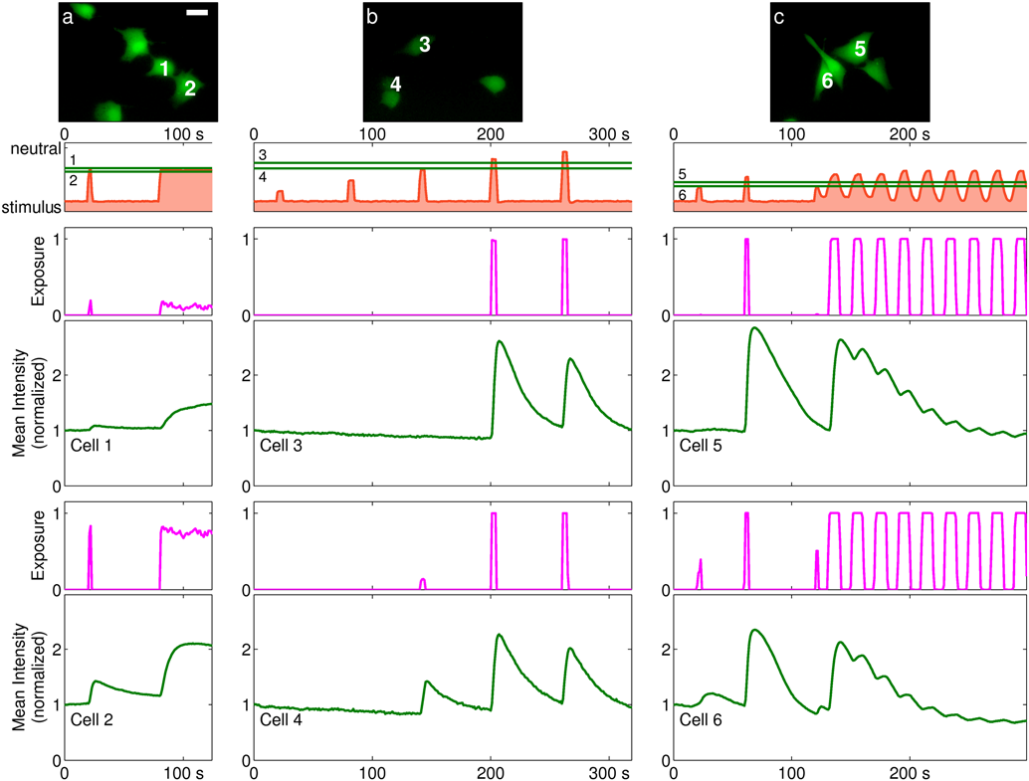
The responses of cells to different exposure signals. Each column of plots presents similar information to Fig. 3 with error regions omitted. Scale bar is 25 µm. (a) Two cells differentially exposed to a short pulse followed by a step-change in exposure. Because of their different latitudes, Cell 1 experiences a sub-maximal exposure, while Cell 2 experiences a near-maximal exposure. (b) Pulses of gradually increasing latitude; see also Supplementary Video S1. (c) Cells exposed to a short pulse followed by periodic stimulation.

A target cell's exposure to stimulus as described above can be thought of as a “chemical signal” which can be used to probe cell behavior. By making the interface follow different reference patterns, it is possible to expose cells to arbitrary time-varying signals, subject to the performance of the pressure regulators. Figure 4 shows a collection of experiments in which cells were exposed to different input signals. Each column in Figure 4 presents information similar to Figure 3: interface position vs. time, followed by estimated exposure and mean fluorescent intensity for each cell.

In Figure 4, column a, a pair of cells experienced a short pulse followed by a step. The interface was positioned at a latitude between the two cells, such that the cell at higher latitude experienced a sub-maximal exposure to stimulus. That cell indicated a gradual increase in 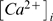. The other cell exhibited a more marked increase, plateau, and a slight decline.

The cells in column b were exposed to short pulses that gradually advanced across the width of the channel. As the cells came under stimulus they demonstrated sudden increases in fluorescent intensity that gradually relaxed after exposure was removed. One cell, stimulated first sub-maximally and then maximally, showed a markedly greater response to the second stimulus. The response morphologies of both cells were similar and repeatable. Subsequent short pulses of comparable size generated smaller responses (see also Supplementary Video S1).

Column c shows cells that were exposed to short pulses followed by a 20-s periodic stimulus. The proportion of each period over which the cells were exposed varied depending on their latitude. The fluorescent intensity signals can be seen to increase during and after the presence of ionophore and decline in its absence, with a short phase delay of around 1–2 s. The magnitude of response was reduced with time, with the initial spike completely relaxed within 180 s, though oscillations in ionophore exposure continued to elicit an oscillatory response.

## Discussion

We have demonstrated the operation of a chemical signal generator which can expose target cells to a stimulus chemical in a spatiotemporally varying manner via control of microfluidic channel inlet pressures. The predictive accuracy of the methodology is governed by the correspondence between the computational model of fluid flow, whose main parameters are the fluid resistances of the inlet and outlet channels, and the actual microfluidic device. If the fluid system is well characterized, then interface position can be inferred from pressure measurements with high accuracy. The spatial precision of our system is 1–2% of outlet channel width and the temporal precision is on the order of 1 s. The precision can be increased by improving the performance of the pressure regulators and the manufacturing accuracy of the microfluidic devices. Better absolute spatial precision can be achieved simply by using narrower channels. Because total flow rate was held constant, shear stresses at the cells were constant before and during each experiment, and so mechanical forces are unlikely to have contributed to any observed fluorescent intensity changes.

Cells responded uniformly to brief 1–3 s pulses of ionomycin, showing a sudden increase in fluorescent intensity during and shortly after the duration of the pulse, and a gradual recovery after the stimulus was withdrawn. Both the duration and the intensity of the stimulus were reflected in the cells' observed responses. Given the low (1 µM) extracellular calcium concentration used in the investigation, the immediate response suggests that the ionophore is mobilizing internal calcium stores to cause a rapid increase in 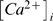, with minimal ionophore-mediated transport from outside the cell [26], [28]. The time for ionomycin to enter the cell appears comparable to the 1-s monitoring interval used in this study. Furthermore, the immediate decline in 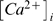 upon removal of the stimulus suggests that the time for ionomycin wash-out is similarly brief. Our intervention thus represents a time- and spatially-controlled intracellular stimulus.

Cells assayed with oscillatory signals (Fig. 2f–j, Fig. 4c) provide further evidence for this interpretation. Cells demonstrated oscillatory responses of identical frequency to the stimulus, suggesting that calcium transport was dominated alternately by ionophore-mediated increase and endogenously motivated decrease of 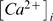 in correlation to the presence of stimulus. The exhaustion of cellular response after repeated or prolonged stimulus (Fig. 2a–e, Fig. 4c) suggests that store depletion is occurring. It may also represent an unexpected mode of regulatory behavior, the analysis of which is now tractable using this new approach.

The time course of fluorescent intensity after or between stimulus events represents the action of the cell's intrinsic mechanisms for regulating 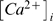. The primary contributors include transmembrane ATPase pumps in both the plasma membrane and the ER membrane and calcium buffers, as well as numerous other mechanisms, both broadly distributed and spatially localized [6]. Store-operated calcium entry can also be expected to contribute, although low extracellular calcium may debilitate this response. Through the use of different stimulus chemicals, different aspects of cellular response to stimuli can be assayed with subcellular precision. For example, the dynamic properties of sarco-endoplasmic reticulum calcium ATPase (SERCA) can be measured quantitatively by comparing pulse responses obtained with and without SERCA inhibitors.

The chemical signal generator can also be used to investigate other calcium regulatory mechanisms of the intracellular calcium store, a highly complex structure which is continuous with the nuclear membrane and extends throughout the cytosol. The store performs complex feedback regulation, sensing its own capacity and responding to changes in the level of cytosolic calcium [7]. Probing it with spatially localized stimuli would allow manipulation of the chemistry of ER subdomains and thus future studies could probe spatially separated regulatory processes. The ability to provide oscillatory signals also introduces the possibility of direct investigation of frequency-encoded regulation pathways, which have been implicated in multiple physiological conditions from cardiac hypertrophy to oocyte fertilization [5].

Because diffusion of solutes across the laminar interface progresses with downstream flow, the sharpness of the chemical gradient at a given longitude can be controlled by adjusting the overall flow rate. Abrupt transitions, such as those demonstrated here, are possible at high flow rates. At lower flow rates the chemical environment can vary gradually from one stream to the other [31]. By adjusting the position of the concentration gradient dynamically through pressure feedback, signals that are continuous in chemical concentration as well as time could be applied to cells or other experimental targets.

Determining the dynamic characteristics of feedback systems is an objective of the engineering discipline of control theory. The introduction of an ionophore can be thought of as a disturbance to a cell's normal homeostatic operation, a common engineering technique used to identify dynamic properties of engineered feedback systems. Though the feedback processes occurring inside cells can be analyzed in a similar fashion to those of human-engineered systems, control theory has seen limited application in cell signaling analysis until recently [2], [32]. Techniques for generating precise time-varying chemical signals, such as the present study, may help make the extensive analytical resources of control theory more available to the study of cell biology.

## Materials and Methods

### Cell Culture

NIH-3T3 Fibroblasts (ATCC, CRL-1658) were cultured in Dulbecco's modified eagle medium (Gibco, 11885) supplemented with glucose (final concentration 4500 mg/L), 10% calf serum, 100 units/mL Penicillin G Sodium, 100 µg/mL Streptomycin Sulfate and 290 µg/mL L-Glutamine (Gibco, 10378), and 10 mM HEPES. The cells were cultured in 5%-CO_2_ at 37°C.

### Microfluidics

Microfluidic devices were fabricated from polydimethylsiloxane (Dow Corning, Sylgard 184) using standard soft-lithography techniques (see [33], Supplementary Material S1). Fluid channels were 50 µm high and the chamber in which experiments were performed was 500 µm wide. Inlet and outlet holes were punched with sharpened snub-nosed 16 ga hypodermic needles.

Devices were sterilized with 70% ethanol and rinsed thoroughly with phosphate-buffered saline solution before use. They were then treated with 20 µg/mL fibronectin (Calbiochem, 341668) for 1 h. Cells were passaged, pelleted through centrifugation, and then resuspended at approximately 10^6^ cells/mL. 20-µL droplets of cells in suspension were placed on the outlet ports of the microfluidic devices, and cells were gently induced into the microfluidic channel through manual application of suction with a micropipetter. Once the presence of a satisfactory number of cells was confirmed, flow was arrested by adding a single 250–400 µL droplet of medium which covered all three ports on the microfluidic device, thus eliminating pressure gradients inside the channels. Cells were allowed to settle and spread out for 60–120 min.

Before the experiment, the cells were loaded with Fluo-4 AM dye (Molecular Probes, F14201) via gravity-driven perfusion. Cells were perfused with medium containing 2 µM dye for 19–20 minutes at 200–400 µL/*h*.

The neutral immersion fluid was a low-calcium formulation of Ringer's solution containing 121 mM NaCl, 2.4 mM K_2_HPO_4_, 0.4 mM KH_2_PO_4_, 1.0 mM MgCl_2_, 1.0 µM CaCl_2_, 10 mM glucose, and 10 mM Hepes, pH adjusted to 7.40–7.43. The stimulus immersion fluid was made of identical Ringer's solution plus 1.0 µM ionomycin (Calbiochem, 407950). Glass syringes containing immersion solutions were loaded into in the pressure regulators and connected to the microfluidic device inlets using polyethylene tubing (Intramedic, B&D 427416).

Devices were operated using custom-designed software written in Matlab *™* (The MathWorks; software available from the authors). The flow rate in the experiment chamber was held roughly constant at 45 µL/min, maximum fluid velocity was 47 mm/s, and shear stress at the walls was 3.75 Pa.

### Pressure Regulation

Fluid flow through the microfluidic devices was controlled with a pair of custom-fabricated feedback-controlled pressure regulators. For a detailed description of their design and operation, please refer to [22] and to Supplementary Material S1. Briefly, glass syringes were held in padded aluminum mounting blocks. Their plungers were actuated by DC servo motors (Maxon Motors) attached to precision leadscrews. Reservoir pressure was measured with a pair of amplified piezoresistive pressure sensors (Honeywell, ASDX015G24R). Pressure regulation was accomplished by custom-fabricated closed-loop servo controllers implemented on microcontrollers (Microchip, PIC18F458). The microcontrollers implemented a Proportional-Integral-Derivative (PID) control law using the pressure signals as feedback. The outputs of the control law were amplified and used to drive the DC motors.

### Data Acquisition and Analysis

Samples loaded into microchannels were placed on an inverted microscope (Zeiss, Axiovert*™* 200) and imaged at 63× magnification using an oil-immersion objective (survey images taken at 10×) Brightfield images at high magnification were acquired using a DIC filter; fluorescent images were excited with a mercury arc-lamp fluorescence source gated by a computer-controlled shutter (Sutter Instrument) and through a FITC filter set (Zeiss). Exposure times were 40–80 ms, and each image stack had uniform exposure. Image acquisition was performed using a Spot Insight*™* camera and software (Diagnostic Instruments). Pressure sensors were calibrated as previously described [22] and uniform calibration values were maintained throughout the investigation. The TTL signal that triggered the shutter was also routed to the pressure regulators. At each image acquisition they logged measurements of reservoir pressure which were used to estimate interface position at the time of imaging. The diffusion coefficient of ionomycin was estimated to be 2.7×10^−10^ m^2^/s based on its molecular weight. Image stacks were processed with NIH ImageJ and Matlab.

## Supporting Information

Supplementary Material S1 Chemical Signal Generator. Supplementary videos; Imaging settings; Fluid dynamics; Pressure Control; Microfluidic Devices.
(0.94 MB PDF)

Video S1 Cells stimulated with pulses of increasing latitude.
(1.44 MB AVI)

Video S2 Cells stimulated with a sinusoidally-varying interface position.
(0.62 MB AVI)
