# Supplementary material for: Probing Cellular Dynamics with a Chemical Signal Generator
Source: PLoS One. 2009 Mar 16;4(3):e4847. doi: 10.1371/journal.pone.0004847 (PMC2653636; doi:10.1371/journal.pone.0004847)
Supplement: Supplementary Material S1 — Chemical Signal Generator. Supplementary videos; Imaging settings; Fluid dynamics; Pressure Control; Microfluidic Devices. (0.94 MB PDF) [file pone.0004847.s001.pdf]

Probing cellular dynamics with a chemical signal generator: Supplementary Materials

Brandon Kuczenski*, Warren C. Ruder, William C. Messner, and Philip R. LeDuc
Carnegie Mellon University

February 18, 2009

Numbered references refer to the supplementary bibliography at the end of this document.

1 Supplementary Videos

The videos included with the submission record fluorescent intensity of cells under the condition of parallel laminar flow. Flow rate was held constant at 45 μL/min and uniform in the period before and during each experiment. Images were captured at 1 s intervals using a 1600×1200 pixel CCD camera using 2×2 binning, in gray-scale with 8 bits of resolution. The output videos are shown at 8 frames/sec (8× speed). In order to facilitate video downloading, the videos have been heavily compressed from the original data. The videos have been post-processed in Matlab to show a horizontal red line that indicates the estimated position of the interface when it is within the field of view, and a red field that indicates the area under stimulus.

Video S1

Supplementary Video S1 shows the data presented in Fig. 1e–i and Fig. 4b in the paper body, beginning at Frame 110 ($t = 0$ s in Fig. 1). The data threshold was adjusted to better fit the dynamic range of the data (old range: 0–255, new range: 0–85).

Video S2

Supplementary Video S2 shows the data presented in paper Fig. 2f–j, beginning at Frame 70.

*Please direct questions about the supplementary materials to this author, bkuczenski@bren.ucsb.edu

Table S-1: Exposure settings for images presented in this paper.

**Figure**	**Objective / Filter**	**Exposure**	**Gain**	**Notes / Post-processing**
Paper Body				
1b–c	10×	10ms	1×	
1d	10× phase-contrast	10ms	1×	
1e	63× DIC	55ms	1×	sharpening (GIMP edge enhancement) and thresholding
1f–i	63× FITC	80ms	4×	2×2 binning; high-threshold adjustment (255→85) (GIMP)
2a	63× FITC	80ms	4×	2×2 binning
2f	63× FITC	60ms	4×	2×2 binning
3a	63× FITC	40ms	4×	2×2 binning
4a	63× FITC	40ms	4×	2×2 binning
4b	63× FITC	80ms	4×	2×2 binning; same experiment as Fig.1f–i
4c	63× FITC	60ms	4×	2×2 binning
Supplementary Videos				
S1	63× FITC	80ms	4×	2×2 binning; high-threshold adjustment (255→85) and overlay added (MATLAB); same experiment as Fig.1f–i, Fig.4b
S2	63× FITC	60ms	4×	2×2 binning; same experiment as Fig.2f; overlay added (MATLAB)

2 Imaging Settings

Included here is a summary of image acquisition data for the images presented in the main body and in the supplementary videos. Table S-1 summarizes exposure settings for all images.

All Images

All images were acquired using an Axiovert™ 200 microscope (Zeiss), a Spot Insight™ camera (Diagnostic Instruments) with 1600×1200 pixel resolution and a pixel size of 7.3 microns. At 10×, this equates to 0.73 microns per pixel. At 63× this equates to 0.117 microns per pixel. The camera has 8 bits of depth resolution (grayscale) and includes hardware gain. All image stacks were acquired at uniform exposure and gain settings.

Fluorescence images were acquired with a Zeiss FITC filter set (Zeiss part number 488010-0000) and a 63× oil-immersion objective. For fluorescence images, 2×2 binning was used, making the effective resolution 800×600 and the effective pixel size 0.235 microns. Gray-scale images are presented in monochrome green.

Image Processing

Images were not processed prior to data extraction. Images were acquired with Diagnostic Instruments Spot software and saved as TIFF multi-image stacks. Stacks were opened in NIH ImageJ software, regions of interest were selected by hand, and mean fluorescent intensity was automatically measured over each region on each frame. The data were then exported to MATLAB, where data presentation was performed. Control regions were selected that were nominally black. Spikes in the control signals were interpreted as camera glitches; those data points were removed (no such points were seen in the data presented). Normalization was performed by dividing data values by the average of the first five data points.

In preparing images for publication, images used in Figure 1 were enhanced for clarity (see Table S-1) in the GNU Image Manipulation Program (GIMP).

3 Fluid Dynamics

The following section is excerpted from [1].

As a consequence of their small scale, fluid flows in microchannels are highly ordered, and therefore are well suited to precise mathematical analysis. The following discussion covers the aspects of fluid behavior that are relevant to the present study. For a complete description of fluid transport in microfluidic systems, please see [2].

In general, fluid flow is characterized by the ratio of inertial to viscous forces. This ratio can be formalized in the Reynolds number, a dimensionless parameter given below:

$$\mathrm{Re} = \frac{\rho U^2}{\mu U/l} = \frac{\rho U l}{\mu} \qquad (1)$$

where ρ and μ represent the fluid's density and kinetic viscosity, respectively; l represents a characteristic length of the flow and U represents a characteristic velocity. The characteristic length of closed channel flow is the hydraulic diameter of the channel, $D_h = 4A/P$, where A is the cross-sectional area and P is the wetted perimeter. Note that, in the case of a circular channel, the hydraulic diameter reduces to the channel diameter. For a rectangular channel whose aspect ratio (width/height) is a, $D_h = 2ha/(a+1)$, or roughly $2h$ for high-aspect-ratio channels. The characteristic velocity is the average volumetric velocity, Q/A, where Q is the volumetric flow rate.

The Reynolds number of a flow determines many of the flow's qualities; in particular, for Reynolds numbers below about 2000 the flow is laminar. Particles in laminar flow are dominated by viscous forces, and

tend to follow smooth, predictable patterns which can be described using a potential equation. Microfluid flows often have Reynolds numbers around 1 or less, and so are highly laminar, sometimes described as "creeping flows."

Channel flow is termed fully-developed when its behavior is consistent with the theoretical behavior of flow under similar conditions in a channel of infinite length [3]. The distance required for a flow to become fully developed is called the entrance length, l_e, and is governed by the Reynolds number. For Reynolds numbers close to 1, the entrance length has been shown to be described by [4]

$$\frac{l_e}{D_h} = \left[(0.631)^{1.6} + (0.0442\mathrm{Re})^{1.6}\right]^{1/1.6} \tag{2}$$

Abrupt changes in channel geometry can disrupt fluid flow, but subsequent flow can be taken as fully developed after the entrance length.

For the present study, with aqueous solutions and characteristic values of $D_h = 100\mu m$ and $U \ll 100mm/s$, Re $\ll$ 10. A typical entrance length is $l_e \ll 100\mu m$. Under this flow regime two distinct transport phenomena become significant: the velocity distribution of advective transport along the axial dimension of the channel, and the diffusive transport of a trace solute in the plane normal to the channel axis.

3.1 Velocity Profile

The equations of fluid motion can be derived by modeling the forces upon and material flows through a differential fluid element (see [3]). They can be stated concisely for Newtonian fluids in the application to the problem of pressure-driven laminar flow in a uniform microfluidic channel by:

$$-\frac{dP}{dz} + \mu\left(\frac{\partial^2}{\partial x^2} + \frac{\partial^2}{\partial y^2}\right)u = 0 \tag{3}$$

which is the governing equation for Stokes flow. Assuming symmetric flow and no slip at the channel walls, taking channel width to be w, height h, and aspect ratio $a = w/h$, the boundary conditions are

$$u(w/2,\cdot) = 0;\quad u(\cdot, h/2) = 0;\quad \left.\frac{du}{dx}\right|_{y=0} = 0;\quad \left.\frac{du}{dy}\right|_{x=0} = 0 \tag{4}$$

We can nondimensionalize the problem by taking $\bar{x} = x/(a\frac{h}{2})$, $\bar{y} = y/(\frac{h}{2})$, and $\bar{u} = 8\mu u/Gh^2$, where

$G \triangleq -dP/dz$. Making these changes, the problem becomes

$$\left(\frac{1}{a}\right)^2 \frac{\partial^2 \bar{u}}{\partial \bar{x}^2} + \frac{\partial^2 \bar{u}}{\partial \bar{y}^2} = -2 \tag{5}$$

with appropriate boundary conditions.

It is easy to show that a particular solution of this equation is $\bar{u}_p(\bar{y}) = 1 - \bar{y}^2$. It is a little more difficult, but well within the capabilities of the reader, to show via separation of variables that the solution to the homogeneous problem is given by

$$\bar{u}_h(\bar{x}, \bar{y}) = -4 \sum_{k=0}^{\infty} \frac{(-1)^k}{\lambda_k^3} \frac{\cosh(\lambda_k a \bar{x})}{\cosh(\lambda_k a)} \cos(\lambda_k \bar{y}) \tag{6}$$

where $\lambda_k = \pi(2k+1)/2$.

Returning u to dimensional form but retaining dimensionless measurements for position, the final equation for axial velocity as a function of $(\bar{x}, \bar{y})$ coordinates in the channel cross-section is

$$u(\bar{x}, \bar{y}) = \frac{Gh^2}{8\mu} \left[1 - \bar{y}^2 - 4 \sum_{k=0}^{\infty} \frac{(-1)^k}{\lambda_k^3} \frac{\cosh(\lambda_k a \bar{x})}{\cosh(\lambda_k a)} \cos(\lambda_k \bar{y}) \right] \tag{7}$$

In channels with wide aspect ratios, the velocity distribution is parabolic in $\bar{y}$ and nearly uniform for $|\bar{x}| \ll 1$. This is consistent with the assumption of Hele-Shaw flow far from the channel walls.

If Equation 7 is integrated along each cross-sectional dimension, the result describes volumetric flow rate.

$$Q = \left(\frac{Gah^4}{12\mu}\right) \left(1 - \frac{6}{a} \sum_{k=0}^{\infty} \lambda_k^{-5} \tanh(\lambda_k a)\right) \tag{8}$$

$$Q = G \cdot \kappa^{-1}; \qquad \kappa = \frac{12\mu}{ah^4} \left(1 - \frac{6}{a} \sum_{k=0}^{\infty} \lambda_k^{-5} \tanh(\lambda_k a)\right)^{-1}$$

Continuity requires that the flow rate remain constant along the length of the channel, and so the parameter κ can be taken to be a fluid resistance per unit length of the channel. Integrating Equation 8 again, this time along the length of the channel, produces an expression relating the pressure drop along the channel to the product of the flow rate and the fluid resistance:

$$GL = -\Delta P = Q\,(\kappa L) \tag{9}$$

This equation can be used for macroscopic modeling of microfluidic device behavior. The formulation in

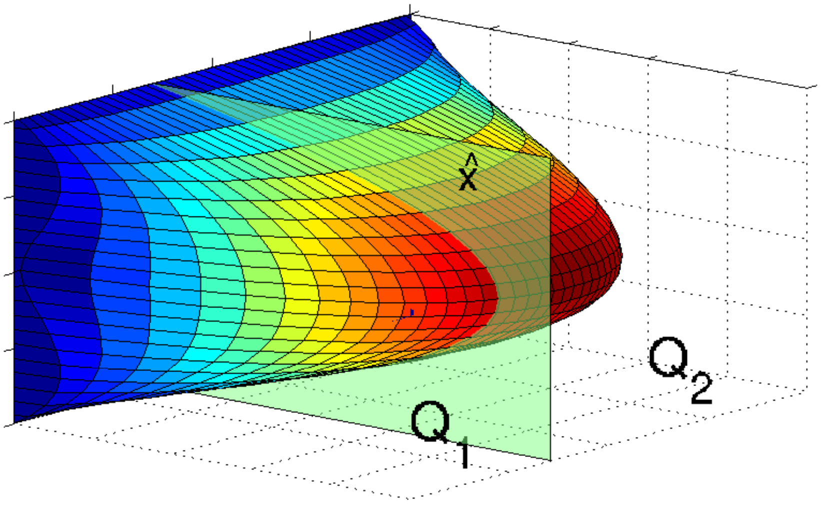

Figure S-1: The two flow volumes created by a laminar interface plane.

Equation 9 is analogous to Ohm's Law for electrical circuits, with fluid pressure replacing electrical potential and fluid flow replacing electric current.

3.2 The Laminar Stream Interface

Consider a planar microfluidic device in which two channels with separate inlets converge to form a third channel with a single outlet. If there is positive flow in both inlet channels, then conservation of mass requires that the flow rate in the outlet channel equals the sum of the two inlet flows. Additionally, preserving the assumptions from the previous section that hold that the fluid pressure is uniform along every cross-section and fluid velocity normal to the channel axis is zero everywhere, we can conclude that a fluid that enters on one side of a microfluidic channel must remain on that side. Consequently, fluids arriving from different inlets will flow alongside each other in parallel streams without mixing, in volumetric proportion according to their relative flow rates. Finally, if the two fluids are of identical density and viscosity, the prior result for the fluid velocity profile (Equation 7) will continue to hold for the multistream flow after the entrance region.

In this condition the two streams will flow as a single fluid, but they will occupy spatially distinct regions of the outlet channel based on their originating inlet channels. The interfaces between different regions will take the form of planes parallel to the channel axis and normal to the plane of the device. The lateral positions of these interfacial planes are determined by the relative flow rates of the streams and can be predicted by integrating the volume under the velocity profile on either side of the plane and equating the

ratio to the ratio of flow rates.

$$\phi = \frac{Q_1}{Q_2} = \frac{\int_{-1}^{\hat{x}} \int_{-1}^{1} \bar{u}(\bar{x},\bar{y}) d\bar{y} d\bar{x}}{\int_{\hat{x}}^{1} \int_{-1}^{1} \bar{u}(\bar{x},\bar{y}) d\bar{y} d\bar{x}} \tag{10}$$

This notion is visualized in Figure S-1. In the case where the flow rates are known, the interface position $\hat{x}$, which varies between -1 and 1, must be determined by integrating $\bar{u}$ inside of an iterative root finding algorithm.

Manipulation of the position of the planar interface thus depends on manipulation of the flow rates of the multiple streams. Flow rate can be related to the forcing pressure at the inlet of the microfluidic device through the fluid resistance of the inlet and outlet channels, using Equation 9:

$$\begin{aligned} P_1 &= \kappa_1 L_1 Q_1 + \kappa_0 L_0 (Q_{sum}) \\ P_2 &= \kappa_2 L_2 Q_2 + \kappa_0 L_0 (Q_{sum}) \end{aligned} \tag{11}$$

where P_k represents the forcing pressure at the kth inlet measured relative to the pressure at the outlet of the microfluidic device (generally atmospheric pressure) and (κ_k, L_k) are the fluid resistance per unit length and channel length of the different channels (0 represents the outlet). R_k is taken to represent the product $\kappa_k L_k$, the fluidic resistance of inlet k.

Equations 11 can be combined to describe the outlet channel flow rate Q_{sum}:

$$Q_{sum} = \frac{\sum P_i/R_i}{1 + R_0 \sum 1/R_i} \tag{12}$$

This shows that the flow rate in the outlet channel can be held constant as long as $\sum P_i/R_i$ is held constant. In the case where all inlets have equal fluidic resistance, the output flow rate is directly proportional to the sum of the inlet pressures, even if the individual pressures vary.

The fractional flow through inlet k can be defined as $\theta_k = Q_k/Q_{sum}$. Equation 12 leads to a statement of θ_k as a function only of forcing pressures and device geometry:

$$\theta_k = \frac{P_k}{R_k} \left(\frac{1 + R_0 \sum 1/R_i}{\sum P_i/R_i} \right) - \frac{R_0}{R_k} \tag{13}$$

When the ratio θ_k becomes zero, flow through the kth inlet channel ceases. This corresponds to the forcing pressure P_k equaling the pressure at the point of confluence. If P_k becomes lower than the confluence pressure, then backflow up the kth inlet will occur, with the backflowing fluid originating from the adjacent inlet streams.

Equations 10–13 can be used to relate the interface position at confluence to macroscopic channel geometry and to inlet forcing pressures. For a fixed geometry, ϕ depends only on the pressures and manifests no dynamic behavior. Thus the interface position near the confluence of the inlet streams at any instant depends only on the pressures. According to this theory, interface position can be modulated as precisely and quickly as the inlet pressures can be modulated. Practically, this approximation is valid as long as the characteristic speeds of the system are much lower than the speed of sound in water, and the characteristic time scales are much longer than the slowest mechanical time constants of the system, i.e. that the system is quasi-steady-state.

Interface position in this discussion refers to the interface at a point immediately after the flow becomes well-developed. Changes in the forcing pressures will manifest immediately at the point of confluence and propagate downstream with a characteristic transport delay based on the fluid velocity. Under typical conditions, this delay is on the order of 10-100 ms for a point 1-2 mm downstream.

3.3 Diffusion Across the Interface

The previous section explored the phenomenon of parallel laminar streams flowing adjacent to one another in a microfluidic device. Although the streams must be identical in macrophysical characteristics in order for the prior results to be meaningful, the streams could still differ in the presence and concentration of trace solutes. In the case that two adjacent streams differ in chemical concentration, their juxtaposition in the outlet channel will produce a sharp concentration gradient across the laminar interface that separates them. Where there is a concentration gradient, there is diffusion. A complete discussion of diffusive transport can be found in [5].

Diffusive transport of trace solutes is governed by Fick's First Law:

$$\mathbf{J_A} = -D\nabla c_A \tag{14}$$

which states that the flux of a diffusing substance A is proportional to the concentration gradient of the substance ∇c_A, with the constant of proportionality being the diffusion coefficient D, and the direction of the flux tending to reduce the gradient. If Equation 14 is combined with the equation of continuity in modeling a differential fluid element, a general transport equation can be written that includes both advection and diffusion:

$$\frac{\partial c_A}{\partial t} + \mathbf{u} \cdot \nabla c_A = \nabla \cdot (D\nabla c_A) + R_A \tag{15}$$

where R_A represents the generation or depletion of substance A by chemical reaction.

Equation 15 can be used to model diffusion across the laminar interface in a microfluidic channel. Microfluid flows in this study can be assumed to be steady state with no velocity components normal to the channel axis and no reaction. The diffusion coefficient can be taken as constant. In addition, under the condition that convective transport downstream is much larger than diffusive transport, diffusion along the axis of the channel can be neglected. The ratio of convective to diffusive transport is measured by the Péclet number,

$$\mathrm{Pe} = \frac{Uh}{D} \tag{16}$$

and diffusion in the axial direction can be neglected when $\mathrm{Pe} \gg 1$, as is the case in this study.

The simplified form of the advection-diffusion equation can then be written:

$$u(x,y)\frac{\partial c_A}{\partial z} = D\left(\frac{\partial^2 c_A}{\partial x^2} + \frac{\partial^2 c_A}{\partial y^2}\right) \tag{17}$$

This formulation can also be nondimensionalized as an aid to analysis. As before, we take $\bar{u} = 8\mu u/Gh^2$ to be the peak velocity at the center of the channel. However, we now want $\bar{x}$ and $\bar{y}$ to be commensurable, because diffusion is equiaxial, so we take $\bar{x} = x/(\frac{h}{2})$ and $\bar{y} = y/(\frac{h}{2})$. Because we are neglecting diffusion in the z direction, we are free to nondimensionalize z against a characteristic downstream length to be determined later, $\bar{z} = z/z^*$. Lastly, chemical concentration of the solute, c_A, is assumed nondimensional, where $c_A = 1$ corresponds to the maximal concentration present in the problem's initial condition.

$$\bar{u}\frac{\partial c_A}{\partial \bar{z}} = \frac{1}{\nu}\left(\frac{\partial^2 c_A}{\partial \bar{x}^2} + \frac{\partial^2 c_A}{\partial \bar{y}^2}\right); \quad \nu = \mathrm{Pe}\cdot\frac{h}{4z^*} \tag{18}$$

We can now select our characteristic length, z^*, to be $\mathrm{Pe}\cdot h/4 = Gh^4/32\mu D$, which renders the problem parameter-free.

The Prototypical Case: One-Dimensional Diffusion

Under the assumptions of a uniform velocity field and solute distribution in the y direction, Equation 18 reduces to the one-dimensional Diffusion equation,

$$\frac{\partial c_A}{\partial \bar{z}} = \frac{\partial^2 c_A}{\partial \bar{x}^2} \tag{19}$$

whose solution, under the initial condition that $c_A|_{\bar{x}<0} = 1$ and $c_A|_{\bar{x}>0} = 0$, is [3]

$$c_A = \frac{1}{2}\left(1 + \mathrm{erf}(-\eta)\right); \quad \eta = \frac{\bar{x}}{2} \cdot (\bar{z})^{-1/2} \tag{20}$$

where erf(x) represents the Gauss Error Function.

Equation 20 accurately describes experimental results measuring diffusion in microchannels, and largely governs the operation of diffusion-based microfluidic molecular sorters and sensors for which variations along the channel's vertical axis are not significant [6].

Two-Dimensional Effects

Because the velocity field in real microchannels is not uniform, Equation 20 is limited in its applicability. The fluid velocity is close to zero near the channel walls, so diffusion is more significant and the diffusion region can be expected to grow more quickly. Published results have suggested that near the walls of a channel, the flow grows with the downstream distance as $\bar{z}^{1/3}$, which would represent a faster rate of growth for $\bar{z} < 1$ [7].

An analytic evaluation of the problem is challenging, but there are two exemplary treatments in Salmon and Adjari [8] and Jiménez [9]. Both take insight from a classic solution by Lévêque (1928; treated in Schlichting [10]) for a problem of heat transfer from a fixed-temperature plate into a laminar film. The solution relies on a change of variables $\hat{y} = 1 - \bar{y}$ to place the origin against the channel wall, with the assumption that the velocity is linear in $\hat{y}$ in a small region near the wall. The transformed problem can be nonparameterized with a pair of new similarity variables which vary with $\bar{z}^{-1/3}$. However, no analytic solution is forthcoming, placing the problem of determining the transverse concentration profile in the domain of numerical methods.

Modeling the Concentration Profile

Numerical models have been used to study the problem and have replicated the observed accelerated broadening of the diffusion region near the walls [8, 9]. These models also find a 1/3-power broadening close to the channel walls. Figure S-2 shows representations of the numerical solution for concentration distribution in cross-sectional planes at distances of $0.01z^*$ and $0.2z^*$ from the confluence, as computed from a finite-difference implementation of Equation 18.

In order to create a simple formulation of chemical concentration near the wall of the channel as a function of lateral distance from the interface plane and distance downstream, we used the numerical model to extract

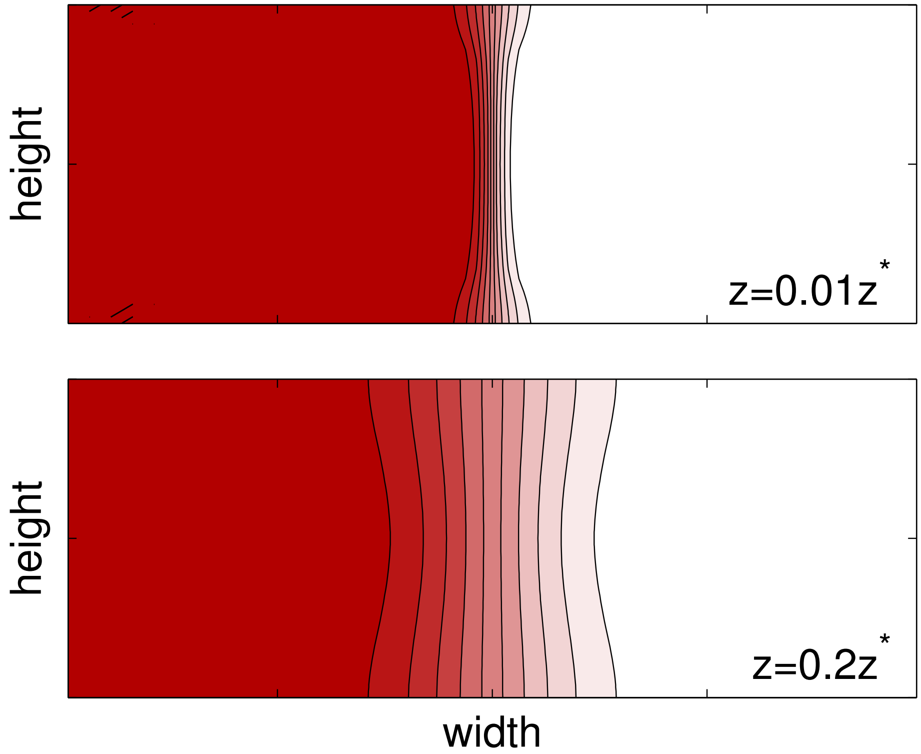

The concentration profile in a plane normal to the axis, in simulation.

Figure S-2: Modeling diffusion across the laminar interface.

contour curves of chemical concentration in a plane just above the bottom of the channel ($\bar{y} \to -1$). We defined the diffusion region δ as the distance from the interface beyond which the concentration was below a threshold value, and made the supposition that δ grew with $\bar{z}^{1/3}$ for $\bar{z} \ll 1$. We sought the values of $f(c_A)$ for the formulation,

$$\frac{\bar{\delta}}{2} = f(c_A) \cdot (\bar{z})^{1/3} \tag{21}$$

where $\bar{\delta} = \delta/(\frac{h}{2})$ and $\bar{z} = z/z^*$. For each of several threshold values r we computed contour curves from the data for $\bar{\delta}(r, \bar{z})$. We used the contours to determine the downstream distance that minimized the error between $\bar{\delta}$ and $\alpha\bar{z}^{1/3}$ for some optimal α. This distance was $\bar{z} = 0.0414$. We then determined the best-fit value for α at each threshold value over the region $\bar{z} \in [0, .0414]$. The results are shown in Figure S-3a, and are well-described by the function

$$\alpha(r) = f(c_A) \approx -\mathrm{erf}^{-1}(2c_A - 1) \tag{22}$$

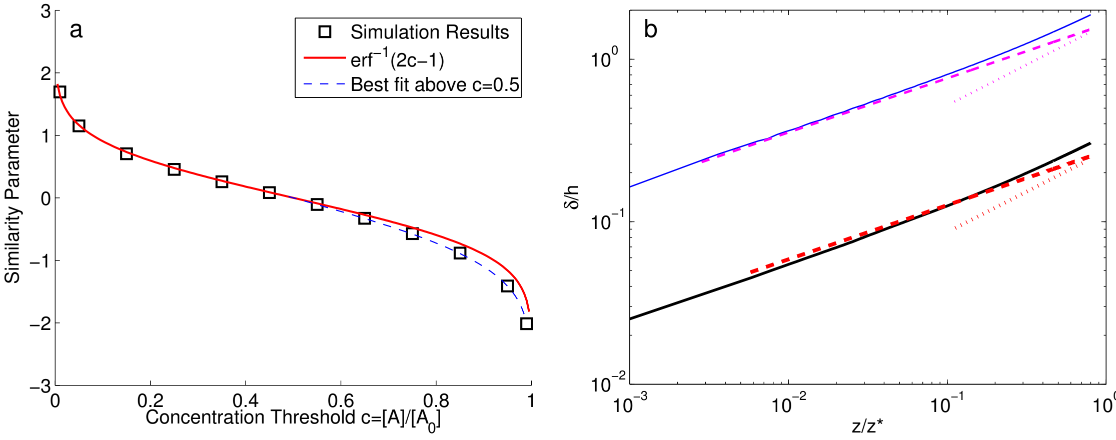

(a) The similarity parameter $\bar{\delta}/\bar{z}^{1/3}$, representing growth of the diffusion region near the wall for threshold concentrations from 0.01–0.99 (squares). The solid line shows a plausible formulation of diffusion region growth. (b) Comparison of the simulated data (solid lines) and the analytic model in Equation 22 (dashed lines) for two different concentration thresholds. The classical 1/2-power model is also shown (dotted lines).

Figure S-3: Numerical simulation of the growth of the diffusion region.

for threshold values below 0.5. For threshold values above 0.5 the best fit curve is

$$f(c_A) \approx -1.217 \cdot \operatorname{erf}^{-1}(2c_A - 1) \tag{23}$$

The concentration profile within the plane as a function of $\bar{\delta}$ can then be found by inverting Equations 21–22.

$$c_A = \frac{1}{2}\left(1 + \operatorname{erf}\left(-\bar{\delta}/2 \cdot (\bar{z})^{-1/3}\right)\right) \tag{24}$$

Equation 24 relates concentration of a dilute species initially present in one lane to the distance downstream $\bar{z}$ and the lateral distance away from the interfacial plane, $\bar{\delta}$. Due to the negative sign in the argument, positive values for $\bar{\delta}$ correspond to regions of lower-than-median concentration.

The 1/3-power assumption is fairly accurate over a large range of values for $\bar{z}$. Figure S-3b shows the comparison of the analytic model (dashed lines) with the simulation (solid lines) for two submedian concentrations of 1% (thin, blue) and 35% (thick, black). The classical 1/2-power fit is also shown (dotted lines). The 1/3-power approximation appears to be accurate up to $\bar{z} \approx 0.1$, beyond which the slope of the contour curves appears to gradually approach the 1/2-power approximation.

3.4 Impingement of Cells on Fluid Flow

The above theory is developed for an ideal fluid channel with uniform rectangular cross-section. Because the application involves cells adhered to the inside wall of the channel, it is important to consider the potential effects of a cell's impingement on fluid flow.

Although fluid flow around cells in microchannels has never been directly imaged, computational models have been constructed to evaluate the shear force exerted on cells and the displacement of fluid flow by cells [11–13]. Upon review, Walker et al. concluded that fluid flow is significantly affected by the presence of cells if the ratio of cell height to channel height is greater than about 0.1 [14], though the shear force on cells is always higher than the shear force predicted at the walls. Measurements of the three-dimensional geometry of adherent fibroblasts using vertical scanning interferometry have found their maximal height to be in the range of 1.5–2.5 microns, with the mean height over the cell area substantially less than that [15].

In the present application, that height corresponds to a maximal cell-height-to-channel-height ratio of 0.03–0.05. Thus, while the presence of cells on the channel surface could affect the velocity profile in the neighborhood of the cells, those effects are unlikely to manifest significantly outside of the immediately surrounding region, due to the cells' low cross-section in comparison to the channel height.

Cells are also unlikely to affect downstream flow. Fluid flow in the present application is strongly laminar ($Re \ll 10$), and because cellular length scales are considered to be within the scope of classical fluid dynamics, deformation of fluid flow can be thought of as smooth and stable (potential flow; see [3]). In a channel of uniform cross section under such conditions, small perturbations are highly unlikely to induce downstream turbulence or eddy currents which would cause mixing (cf. [16, 17]).

Further investigation may be called for to determine the extent of deformation induced by adherent cells. However, existing research suggests that it is not significant to the present work.

4 Pressure Control

The following section is excerpted from [1].

The objective of this research was to control the chemical concentration in a region of fluid near a point on the interior surface of a microfluidic device using the principles developed in Section 3. Because flow rate depends strongly on forcing pressure, the strategy was chosen to perform closed-loop feedback regulation of static pressure using two independent pressure regulators which would operate synchronously via a computer interface.

Pressure sensor feedback is integral to this strategy. The biggest shortcoming of available displacement-

driven flow control devices is the lack of a means to measure real flow rate. The theory developed in Section 3.2 indicates that the position of the interfacial plane in a microfluidic channel can be estimated based solely on the measurement of forcing pressures, assuming the macro-scale characteristics of the device are known. This implies that chemical concentration at a target region can be estimated regardless of the performance of the pressure regulators. This condition also implies that conventional, open-loop displacement-driven pumps can be used for the same purpose as the custom apparatus used in this investigation. However, the performance of these systems may be inferior in precision or responsiveness to the device described here.

The design concept called for a means of regulating fluid pressure in a reservoir with high speed and precision. The design, motivated by existing displacement-driven syringe pumps, was to use a high-performance DC motor to exert force on a syringe plunger in response to pressure readings from a sensor affixed downstream of the syringe mouth. The chassis was designed to minimize friction, backlash and other nonlinearities as much as possible in order to improve performance. The electrical hardware was chosen to be simple and easily manufacturable, using a small number of commonly-available components.

4.1 Mechanical Subsystem

Figure S-4 shows a number of photographs of the mechanical design. The syringe barrel itself is mounted in a V-shaped channel via a cross-brace attached with thumbscrews. The channel is padded with adhesive foam rubber to permit some flexibility and prevent binding. It was expected that a small amount of compliance would permit the syringe and plunger to settle into a low-friction configuration easily. The syringe axis is designed to be parallel to, and nearly collinear with, the motor axis.

The plunger carriage contains four press-fit brass bushings which slide on a pair of precision 1/4-inch (6.35-mm) steel rods. The two main components of the carriage are the plunger grip, in the front, and the lead-nut mount, in the rear. They are attached via a pair of thin threaded rods. This means of attachment was designed to be tuned carefully during assembly to avoid binding.

The leadscrew and mating lead-nut were chosen for their minimal backlash and rotating friction. The leadscrew is fastened to the motor shaft using a three-axis flexible coupling which was capable of absorbing translational and angular shaft mis-alignment while still retaining rotational stiffness. The motor is mounted to the chassis using flathead screws in countersunk holes, which provide maximal positioning precision.

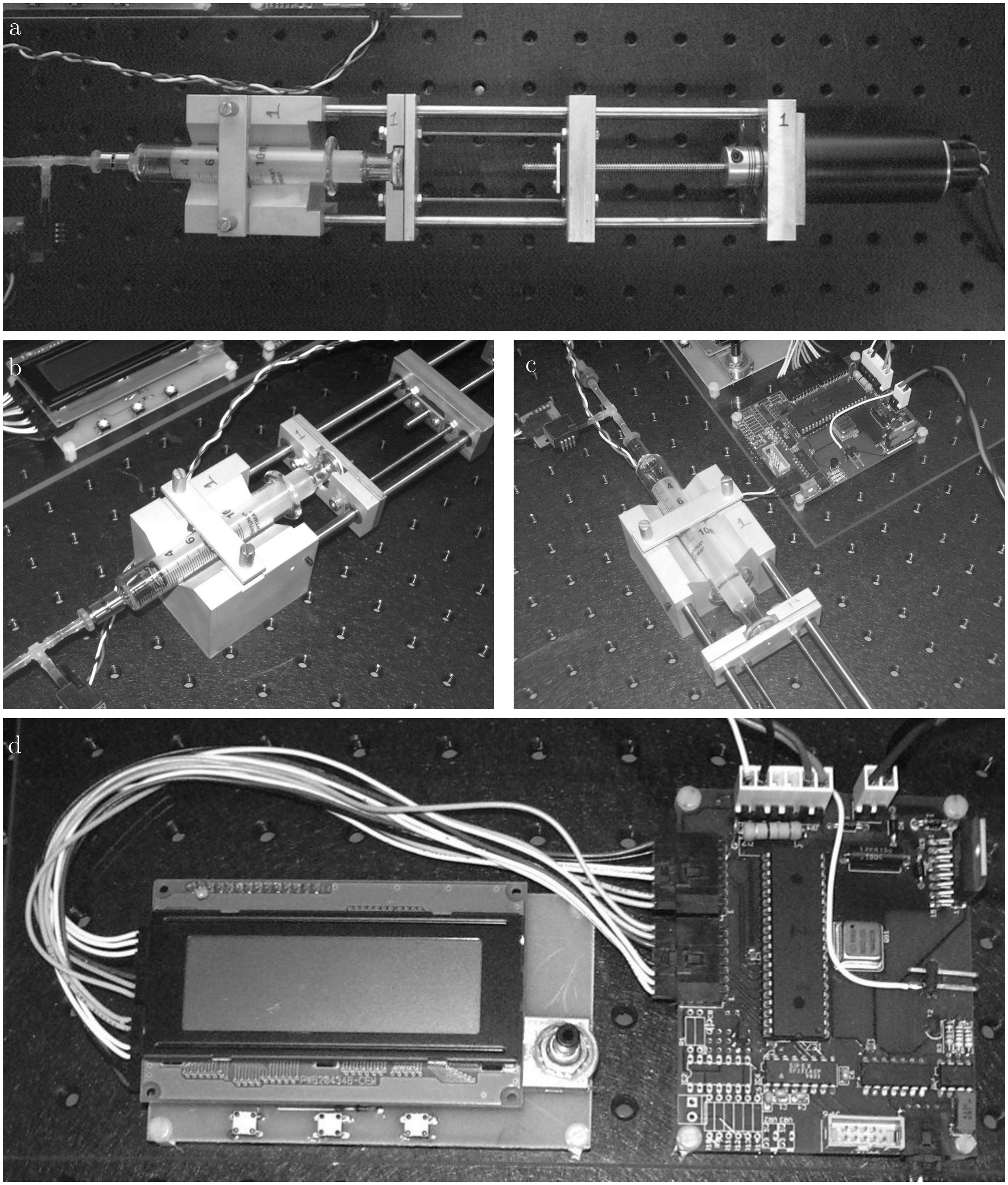

Figure S-4: Photographs of the experimental apparatus.

4.2 Electrical Subsystem

Figure S-4d shows a photograph of the operating electronics for the device. The design goal was a "single-chip solution" which used commonly available components and a single low-cost, multi-purpose processor to handle all functions of the device. These include Analog-to-Digital conversion, user interface via hardware and RS-232 communication, control signal computation, and compensator output via pulse-width modulation.

The PIC 18F series of microcontrollers from Microchip Technology Inc. was selected because of its multiple integrated features and low cost. In addition to the processor, the electrical system includes an RS-232 level converter, some analog circuitry, an L298 H-bridge amplifier and connecting logic. The device also required peripheral hardware including the pressure sensor, LCD screen (Hitachi HD44780-based; 20x4 character display), three pushbuttons, an optical quadrature encoder used as an input knob, and a three-output power supply ($+/-12$ volts, $+5$ volts, and ground).

4.3 Control System Design

Figure S-5 documents the features of the control system. Inputs are the reference signal from the user and the sensor reading from the 10-bit Analog to Digital (A/D) converter. The output is the 10-bit PWM signal to the motor amplifier. The compensator is oblivious to sensor calibration and to real-world measurements. Reference signals are translated into the units of A/D counts and compared directly to the input from the sensor.

With the exception of the servo frequency, which can presently only be adjusted when the code is compiled, all parameters can be adjusted during operation using the serial communication interface.

4.3.1 Reference Signal

The operator selects from three different reference sources: the internal reference, set from the hardware interface; the reference signal from the serial port; or the analog signal coming in on the second A/D channel, AD1.

The reference signal can be altered in two ways. First, the signal's velocity can be limited parametrically to between 4 and 1000 A/D counts per second, permitting step inputs from the operator to be interpreted as continuous linear curves. Second, a parametric sine wave generator, implemented on the PIC, can add a sinusoid to the reference. The sine wave generator has 8 bits of resolution and can produce signals with amplitudes up to 1/4 the maximum reference (peak-to-peak) and periods of integer multiples of 800 ms, up to 204.8 seconds.

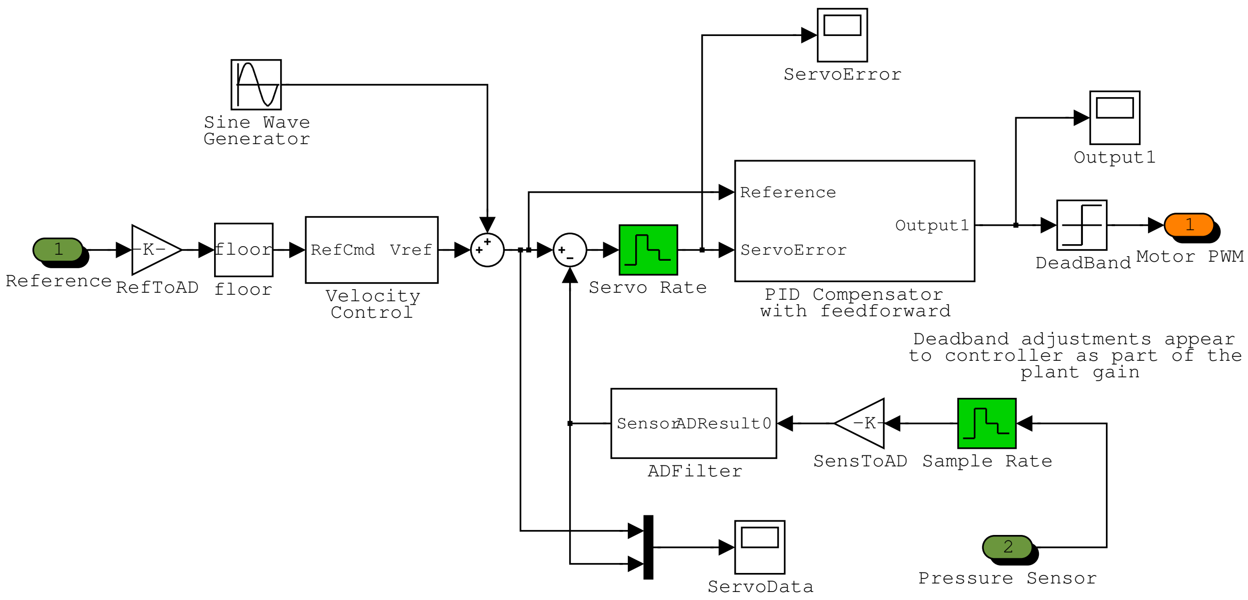

Figure S-5: Design features of the control system.

4.3.2 Feedback Signal

The feedback signal is always read from the first A/D converter channel, AD0. The data acquisition routine subtracts a configurable zero-offset value from the A/D converter result upon receiving it. After zero adjustment, the A/D result is passed through a tunable first-order low-pass infinite impulse response (IIR) filter to reduce noise. The output of the filter is subsequently used as the sensor reading. The filter's transfer function is

$$\frac{\texttt{Sensor}[z]}{\texttt{ADResult0}[z]} = \frac{z \cdot \frac{K_{IIR}}{256}}{z - (1 - \frac{K_{IIR}}{256})} \tag{25}$$

where K_{IIR} is an integer from 0 to 255. For small values of K_{IIR}, this is roughly equivalent to the continuous-time filter $G(s) = \frac{1}{\tau s+1}$, where $\tau = -T_s/\ln(\frac{K_{IIR}}{256})$.

The compensator can be configured to run in an open-loop configuration, where the feedback signal is ignored and a signed reference signal is input directly to the compensator.

4.3.3 Linear Compensator

The control system includes a proportional-integral-derivative (PID) compensator with 32 bits of internal numerical resolution. In order to address the dependence of PID gains on servo frequency, the servo frequency can be set at compile-time to any power-of-two division of the 1-kHz sampling frequency.

The compensator also implements reference feedforward, which is a strategy for predicting the desirable

motor output based only on the reference signal. Because feedforward computation does not depend on the sensor reading, it does not affect the stability of the feedback loop. However, it can have substantial effects on compensator performance. The feedforward term is equal to the reference signal multiplied by a constant programmable gain.

The compensator's internal accumulator retains 32 bits of resolution, but only the most significant 11 bits (10-bit PWM signal plus sign) are used in the output.

4.3.4 Diagnostics and Data Logging

The control software includes a data collection routine which can be used for experimental data as well as device diagnostics. Data collection can be triggered by an internal timer, by an external voltage signal, or via a command received over serial communication. When a trigger event occurs, the routine records a data point. By default, one data point comprises the Reference signal, the Sensor feedback signal, and a configurable third memory register which can log any operational variable. The current implementation has a memory capacity of 768 bytes, or 128 data points of three 16-bit signals. The routine can also be configured to log a single signal for up to 384 points. Data can then be extracted to a computer over the serial port.

This facility enables verification of the control software as well as pressure data logging during an experiment. For fluorescent microscopy, the device can be setup to trigger from the same signal used to open the fluorescent excitation shutter.

5 Microfluidic Devices

The following section is excerpted from [1].

5.1 Microfluidic Device Design

The microfluidic devices were designed to be planar devices with piecewise uniform rectangular cross-section, 50 microns in height. The experiment chamber was designed to be 500 microns wide, affording a large aspect ratio, and 10 mm in length. The operating point was selected to be around 150 kPa/m in the experiment chamber, resulting in a fluid velocity of 47 mm/s, flow rate of about 45 μL per minute, and wall shear force of 3.75 Pa. Under these conditions, the transport delay at the end of the experiment chamber is on the order of 0.25 s and the width of the diffusion region there is around 22 microns.

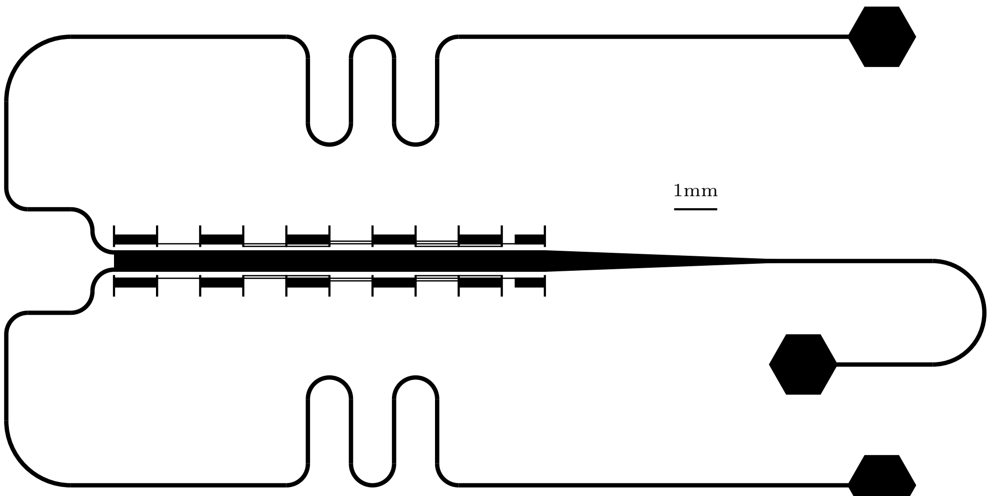

Figure S-6: The microfluidic device design used in cell stimulation experiments.

We chose a two-inlet design for the network in order to deliver one agonist and one neutral stream. The resistance ratio R_i/R_o was chosen to be around 2.3 based on the pressure controllers' ideal operable range of roughly 15–50 kPa. The final channel design, shown in Figure S-6, had an estimated inlet resistance of 47.7 kPa/(μL/s) and outlet resistance of 22.4 kPa/(μL/s). Along the side of the experiment chamber is a coded hash mark strip intended to facilitate easy measurement of longitude.

5.2 Microfluidic Device Manufacture

Microfluidic devices were manufactured according to the practices described in [18]. The designs were laid out in LaTeX and printed to a high-resolution (5080 dpi; 200 dots per mm) negative transparency mask (PageWorks). The masks were used to UV-expose a 50-micron layer of SU8-50 photoresist, which was then hardened, developed and rinsed.

PDMS was mixed with its curing agent in a 10:1 ratio by weight, poured over the wafer and then placed in a vacuum chamber for 20–40 minutes to remove air bubbles. Next it was cured on a hot plate for 1 hour. Molded polymer slabs were cut out with a scalpel. Inlet and outlet ports were punctured using a sharpened flat-end 16-gauge hypodermic needle. The bonding surface was cleaned of lint with Scotch tape and the slabs were placed face-up in an oxygen-plasma oven (Harrick Plasma, PDC-32G). 22×40 mm #1 coverslips were cleaned and rinsed thoroughly, dried with compressed nitrogen, and placed in the plasma oven. The polymer slabs and coverglasses were cleaned in plasma for 60–90s and then bonded together immediately. Finally, they were stored in an oven at 70° C to enhance adhesion.

5.3 Sensor Calibration and Device Precision

The results our previous work demonstrate that the theory presented in Section 3.2 accurately predicts the relationship between static reservoir pressure and laminar interface position [19]. However, interface position determined by Equation 13 depends on forcing pressures and fluid resistances, and so accurate positioning of the interface requires accurate measurements of pressure and resistance. The initial calibration process for the pressure sensors was limited in accuracy by the practical challenge of producing a water column of appreciable height, combined with the problem of noise at the sensor input. The nominal resistance ratio of the microfluidic devices depended on the devices' physical dimensions and characteristics matching their ideal design, when in fact the actual devices seemed to perform somewhat differently. The data processing steps described in [19] aimed to extract accurate gain values for the pressure sensors and to determine the effective resistance ratio, R_i/R_0 of the channels.

In fact, the calibration values extracted from the data can be accurate only in ratio to one another, since there was no measurement that 'anchored' the calibration values to an actual pressure, aside from the initial calibration. Furthermore, the ratio of inlet to outlet resistances fails to account for the possibility of differences in the fluidic resistance of the two inlets from each other. As a result, beyond a merely consistent trend in sensor calibration values and outlet resistance corrections, it was unclear at the conclusion of the above study whether the data processing steps described would permit an experimenter to predict interface position on a freshly-manufactured, uncalibrated microfluidic device.

To answer the question of whether static pressure measurement could consistently predict interface position without requiring a calibration step for every channel, we tested a collection of nominally identical microfluidic devices ($n = 12$) that were in fact manufactured from a variety of distinct molds on two separate wafers. For each device we repeated the analysis described in [19], using the same sensors for all tests and obtaining best-fit values for sensor calibration and effective outlet resistance. We then took the average sensor gain values and effective outlet resistance from the first five tests, and applied them uniformly to the data in all twelve tests. We measured the variance between the interface positions observed and those predicted using the uniform calibration.

The results are shown in Figure S-7. Molds A1–A5 were all on a single wafer, and the data set includes two tests on each. Molds B1–B2 were on a separate wafer and have one test each. The average of the variance, across all tests, is 0.036 dimensionless latitude units, indicated by the dashed line in Figure S-7. This corresponds to $\pm 1.8\%$ of the channel width, or roughly ± 9 microns. The data suggest that the magnitude of the error may be correlated with the specific mold used, and that better characterizing each mold may

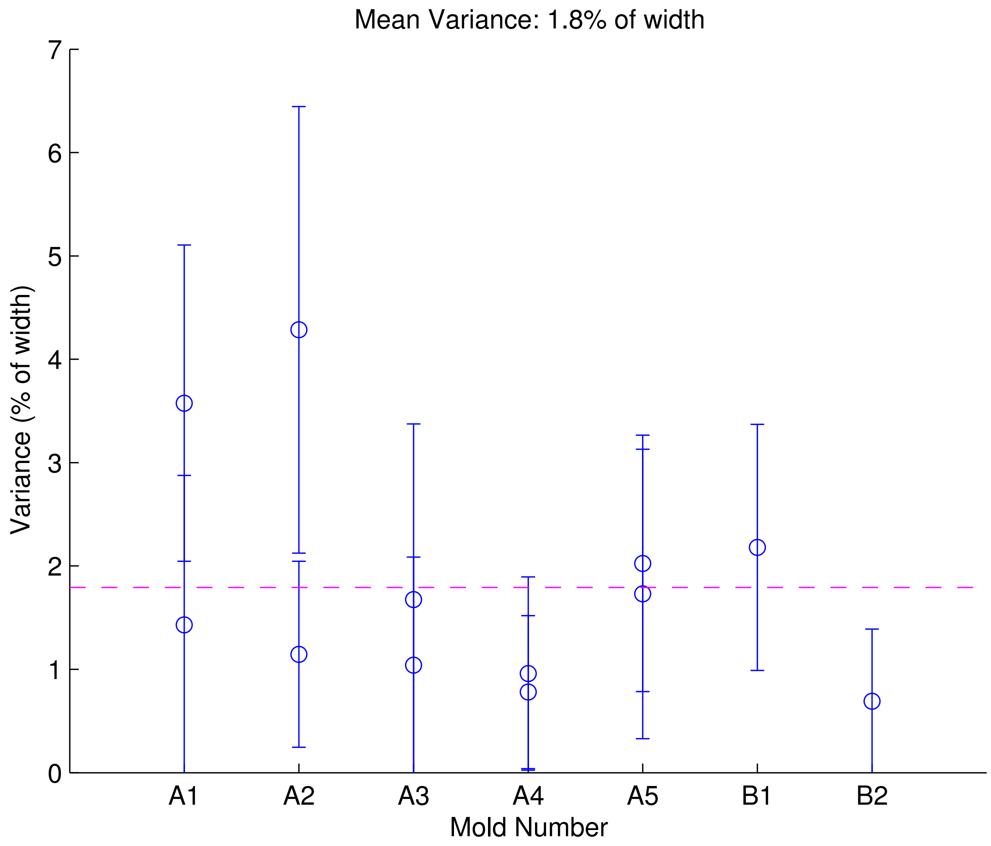

Figure S-7: Mean error in interface position under uniform calibration.

provide improved precision.

Sources of Error

The above procedure makes use of two calibration processes which each manifest a separate source of error. Assuming the pressure sensors themselves maintain a consistent calibration, the sensor calibration step adjusts for discrepancies between the nominally equi-resistive inlets. These discrepancies could be caused by manufacturing defects in the channels or in the particular microfluidic device under test, and may also represent calibration drift of the sensors.

The "effective outlet" step adjusts the slope of the data on the $\Pi - x$ curve by accounting for defects in outlet resistance or in the ratio of mean inlet to outlet resistance. In every recorded test, the effective outlet resistance was larger than the nominal value, suggesting that a systematic error in analysis results in an artifically low predicted value for outlet resistance. A possible explanation is that inlet channels, which are maintained at higher pressures during operation, deform under pressure from the fluid, resulting

in lower inlet resistances and an apparently higher effective outlet resistance. However, it is unlikely that this accounts for the full extent of the observed change, on the order of a 15–25% increase in effective outlet length, and as yet unidentified factors may affect fluid flow. Fortunately, the effective outlet length seems to be consistent across devices for a given design.

Error could be reduced, and positioning accuracy improved, by using more carefully manufactured channels. Soft lithography with low-melting-point polymers is convenient and quick, but may lack the precision required for high-accuracy applications. In the case where tighter accuracy is required, machined microfluidic devices, or devices manufactured out of a hard material or using hard photolithographic techniques, may provide improved performance.[1]

[1] See [20] for a review of manufacturing techniques for microfluidic devices.

References

1. Kuczenski, B. *Probing biological systems using a microfluidic chemical signal generator*. Ph.D. thesis, Carnegie Mellon University (2008). Online. Available: http://leduc.me.cmu.edu/~bkuczens/kuczenski-dissertation.pdf.

2. Tabeling, P. & Chang, S. *Introduction to Microfluidics* (Oxford University Press, 2005).

3. Bird, R.B., Stewart, W. & Lightfoot, E.N. *Transport Phenomena* (Wiley, New York, NY, 1960).

4. Durst, F., Ray, S., Unsal, B. & Bayoumi, O.A. The development lengths of laminar pipe and channel flows. *Journal of Fluids Engineering-Transactions of the ASME* **127**, 1154–1160 (2005).

5. Cussler, E.L. *Diffusion: Mass Transfer in Fluid Systems* (Cambridge University Press, 1997), 2nd ed. ed.

6. Kamholz, A.E., Schilling, E.A. & Yager, P. Optical Measurement of Transverse Molecular Diffusion in a Microchannel. *Biophys. J.* **80**, 1967–1972 (2001).

7. Ismagilov, R.F. *et al.* Experimental and theoretical scaling laws for transverse diffusive broadening in two-phase laminar flows in microchannels. *Appl. Phys. Lett.* **76**, 2376–2378 (2000).

8. Salmon, J.B. & Adjari, A. Transverse transport of solutes between co-flowing pressure-driven streams for microfluidic studies of diffusion/reaction processes. *J. Appl. Phys.* **101** (2007). Art. 074902.

9. Jimenez, J. The growth of a mixing layer in a laminar channel. *J. Fluid Mech.* **535**, 245–254 (2005).

10. Schlichting, H. *Boundary-Layer Theory* (McGraw-Hill, New York, NY, 1979), 7th ed. ed. Tr. J. Kestin.

11. Donald P. Gaver, I. & Kute, S.M. A theoretical model study of the influence of fluid stresses on a cell adhering to a microchannel wall. *Biophys. J.* **75**, 721–733 (1998).

12. Sugihara-Seki, M. Flow around cells adhered to a microvessel wall. i. fluid stresses and forces acting on the cells. *Biorheology* **37**, 341–359 (2000).

13. Sugihara-Seki, M. Flow around cells adhered to a microvessel wall. ii. comparison to flow around adherent cells in channel flow. *Biorheology* **38**, 3–13 (2001).

14. Walker, G.M., Zeringue, H.C. & Beebe, D.J. Microenvironment design considerations for cellular scale studies. *Lab Chip* **4**, 91–97 (2004).

15. Revell, C.M. *et al.* Characterization of fibroblast morphology on bioactive surfaces using vertical scanning interferometry. *Matrix Biol.* **25**, 523–533 (2006).

16. Wang, H., Iovenitti, P., Harvey, E. & Masood, S. Numerical investigation of mixing in microchannels with patterned grooves. *J. Micromech. Microeng.* **13**, 801–808 (2003).

17. Nguyen, N.T. & Wu, Z. Micromixers—a review. *J. Micromech. Microeng.* **15**, R1–R16 (2005).

18. Duffy, D.C., McDonald, J.C., Schueller, O.J.A. & Whitesides, G.M. Rapid prototyping of microfluidic systems in poly(dimethylsiloxane). *Anal. Chem.* **70**, 4974–4984 (1998).

19. Kuczenski, B., LeDuc, P.R. & Messner, W.C. Pressure-driven spatiotemporal control of the laminar flow interface in a microfluidic network. *Lab Chip* **7**, 647–649 (2007).

20. Nguyen, N.T. & Werely, S.T. *Fundamentals and Applications of Microfluidics* (Artech House, Norwood, MA, 2002).
